# Learning from deaths: Parents’ Active Role and ENgagement in The review of their Stillbirth/perinatal death (the PARENTS 1 study)

**DOI:** 10.1186/s12884-017-1509-z

**Published:** 2017-10-02

**Authors:** Danya Bakhbakhi, Dimitrios Siassakos, Christy Burden, Ffion Jones, Freya Yoward, Maggie Redshaw, Samantha Murphy, Claire Storey

**Affiliations:** 1 Centre for Academic Women’s Health, University of Bristol, Chilterns, Women’s Health, Southmead Hospital, Bristol, BS10 5NB UK; 2 University of Bristol, Bristol, UK; 3 Policy Research Unit in Maternal Health and Care, National Perinatal Epidemiology Unit (NPEU), University of Oxford, Oxford, UK; 4 Faculty of Health and Social Care, The Open University, Milton Keynes, UK; 5 International Stillbirth Alliance, Bristol, UK

## Abstract

**Background:**

Following a perinatal death, a formal standardised multi-disciplinary review should take place, to learn from the death of a baby and facilitate improvements in future care. It has been recommended that bereaved parents should be offered the opportunity to give feedback on the care they have received and integrate this feedback into the perinatal mortality review process. However, the MBRRACE-UK Perinatal Confidential Enquiry (2015) found that only one in 20 cases parental concerns were included in the review. Although guidance suggests parental opinion should be sought, little evidence exists on how this may be incorporated into the perinatal mortality review process. The purpose of the PARENTS study was to investigate bereaved parents’ views on involvement in the perinatal mortality review process.

**Methods:**

A semi-structured focus group of 11 bereaved parents was conducted in South West England. A purposive sampling technique was utilised to recruit a diverse sample of women and their partners who had experienced a perinatal death more than 6 months prior to the study. A six-stage thematic analysis was followed to explore parental perceptions and expectations of the perinatal mortality review process.

**Results:**

Four over-arching themes emerged from the analysis: transparency; flexibility combined with specificity; inclusivity; and a positive approach. It was evident that the majority of parents were supportive of their involvement in the perinatal mortality review process and they wanted to know the outcome of the meeting. It emerged that an individualised approach should be taken to allow flexibility on when and how they could contribute to the process. The emotional aspects of care should be considered as well as the clinical care. Parents identified that the whole care pathway should be examined during the review including antenatal, postnatal, and neonatal and community based care. They agreed that there should be an opportunity for parents to give feedback on both good and poor aspects of their care.

**Conclusion:**

Parents were unaware that a review of their baby’s death took place in the hospital. Parental involvement in the perinatal mortality review process would promote an open culture in the healthcare system and learning from adverse events including deaths. Further research should focus on designing and evaluating a perinatal mortality review process where parental feedback will be integral.

**Electronic supplementary material:**

The online version of this article (doi: 10.1186/s12884-017-1509-z) contains supplementary material, which is available to authorized users.

## Background

In the UK, over 5000 babies per year die before or shortly after birth (stillbirths and neonatal death) [1]. The death of a baby can result in a wide range of negative psychological symptoms for parents, wider families and staff [2, 3]. Furthermore, the death of a child has long-lasting detrimental effects on family relationships, finance, and employment, which may result in increased use of health services, stigmatisation and dissociation from society [2, 3]. Negative psychological symptoms may continue through subsequent pregnancies and can impact on maternal bonding relationships with subsequent children and siblings [2–4]. The cost of perinatal deaths for women, their families, the NHS, and society is therefore significant, and likely underestimated.

It is urgent to ameliorate this cost and impact of perinatal death. Indeed, in 2012, The UK Department of Health (DoH) established a Perinatal Mortality Task and Finish Group to improve the review process that takes place in each hospital following the death of a baby either before or shortly after birth. The DoH task group recommended a comprehensive and robust review of all losses from 22 weeks gestation until 28 days after birth, which fits into three of the five domains of the NHS Outcomes Framework 2013–2014 [5]. Furthermore, the DoH task group and the recent Morecambe Bay Report have recommended that there should be scope for parental input into the process from the beginning [6]. This is in line with the Francis inquiry which recommended the need for a consistent culture of openness and candour in the NHS, so that errors can be addressed and lessons learnt [7]; and the MBRRACE-UK Confidential Enquiry, which recommended that parents’ perspectives on their care should be included in the standardised multidisciplinary review of their perinatal death, and the results of the review should be shared with parents [8].

However, the perinatal mortality review (PNMR) process is inconsistent across the UK, and rarely includes formal involvement of bereaved parents. In the MBRRACE-UK Confidential Enquiry only 6 out of 133 (5%) cases had documented evidence that parents’ concerns were included in the review. The results of the internal review were only reported to 12 sets of parents [9]. There is urgent need to understand whether and how parents could be involved in PNMR, before we can develop, test and implement a national PNMR process that involves parents. With this study, we sought first, to investigate bereaved parents’ views on the current PNMR process in South West England, and how it could be improved with parental input.

## Methods

### Study design and context

This is a report of a semi-structured focus group discussion of bereaved parents with diverse experience of pregnancy neonatal loss and their views about parental involvement in the PNMR process. This study was supported by the Stillbirth and Neonatal Death Charity (Sands) and the University of Bristol. The topic guide covered four main areas including thoughts on the current PNMR process; views on how could parents contribute and improve the PNMR process; what would be the best method of feedback following PNMR process and views about an initial letter that would be sent to parents following their pregnancy or neonatal loss from Sands and Department of Health.

### Research participants and sampling

The focus group discussion was conducted in 2015 in South West England. We sampled women and their partners who had a lived personal experience of a perinatal death more than 6 months prior to the study. A purposive sampling technique was used to recruit a diverse sample of women and their partners who had a range of experience of perinatal death. Participants were identified via a key informant who is a bereaved parent and co-investigator (Storey), the local Bristol Sands support group, and the International Stillbirth Alliance. Recruitment took place after the co-investigator had telephone called or emailed and enquired whether they or others might be interested. Following this, study information was sent out to interested contacts and they subsequently decided whether to participate or not. Participants were recruited if they had experienced a mid-trimester loss (from 12 to 24 weeks of gestation), a termination of pregnancy for congenital abnormality, a stillbirth (between 24 and 42 weeks of gestation), or a neonatal death (from birth until 28 days postnatal). Further demographic information about participants has not been disclosed in this report as we do not want to compromise anonymity due to the relatively small numbers and geographical area have recruited from.

### Ethics and consent

This study had ethical approval from the University of Bristol Faculty of Medicine and Dentistry Committee for Research Ethics (Reference 131,452 (11262), 17 October 2014). Written consent was obtained from all participants of the study.

### Data collection

The focus group discussion took place in a small private conference room in a neutral venue outside of hospital premises. Participants were given an information sheet prior to the study and written consent was obtained from those who wished to participate. A male consultant obstetrician and clinical researcher (Siassakos) experienced at qualitative research with bereaved parents, conducted the focus group discussion and guided participants through the session, together with a female co-investigator with personal experience of perinatal death (Storey) and of supporting bereaved parents, in case any participant experienced any emotional difficulties during the focus group discussion. The overall aim of the focus group discussion was to explore parental perceptions and expectations of the perinatal mortality review process; how they would like to contribute to this process and how as parents would like to receive feedback. The focus group discussion lasted two and a half hours and followed a specified schedule (see Additional file 1); an introduction and scene setting; discussion of current process; improvements and thoughts on parental involvement; receiving feedback; thoughts about a preformatted invite letter; and summarisation. Two female medical student co-facilitators (Yoward, Jones) were present to moderate the focus group discussion, keep field notes and produce an observational log of participant behaviour. These notes were used to help triangulate codes in the data analysis of the transcript. The focus group discussion was audio-recorded with a digital recorder.

### Data analysis

The data were fully transcribed and analysed after the focus group discussion. A summary of the transcripts was sent to participants. Two researchers (Siassakos, Storey) carried out coding and identified key themes within the transcript. The thematic analysis followed a six-stage process: familiarisation with the data; generation of initial codes; searching for themes; reviewing themes; defining themes, and naming themes [10]. An interim analysis was performed with input from the wider study group to establish emerging themes. A final report was produced adhering to the Consolidated Criteria for Reporting Qualitative Research (COREQ) checklist for qualitative interviews and focus group discussions [11].

### Patient involvement

From the outset, a bereaved parent has been involved in all aspects of study including focus group discussion design, development of study documents for recruitment and consenting participants, ethical approval, the project advisory board, analysis and interpretation of data and co-authorship of this manuscript [12].

## Results

Eleven participants were recruited into the focus group discussion (three participants refused to participate), which took place in November 2015. The age range of participants was early 20s to mid-40s. There were 8 women and 3 male partners in attendance. Participants had experienced the death of a baby at various gestations including mid-trimester miscarriage (early stillbirth), termination for congenital anomaly, late stillbirth, early and late neonatal death, twin and singleton pregnancies. Following thematic analysis four key themes emerged from coding the data including the need for: transparency; a flexible yet specific process; inclusivity and a positive process. See Additional file 2 for additional parental quotes.

### Transparency

Most participants were unaware that a formal perinatal mortality review process took place after the death of a baby. Several had postnatal appointments with consultants however they were not made aware of a review meeting taking place.

*‘I don’t know what was discussed about my son, and after all he is my son, I should know what you know what’s happened? What’s been talked about him? And I mean you know what’s been talked about with your child at school so why should you not know what’s been talked about your son even after he’s dead.’*

Participants wanted a clear process and reassurance that the same thing would not happen again to other parents if a preventable cause was found. They wanted to know what changes will be implemented following their loss and they wanted documented accountability in the process.

*‘What will change as a result of my son dying so that another child doesn’t die of the same thing?’*

All participants wanted to know when the perinatal mortality review process was taking place and to have the lessons learned clearly communicated to them. Parents communicated varied levels of dissatisfaction with the current process.

*‘To me there is something fundamentally wrong, at no point did someone give us a piece of paper saying we’re really sorry your child has died and this is how we investigate it.’*

As discussion developed within the focus group one participant suggested *“an open door”* policy whereby parents were invited to contribute to the review if they wished. The group appeared in agreement with the suggestion and they all felt that parents should be asked specifically if they wished to be involved and wanted to know the outcome of the perinatal mortality review process.

### Flexible yet specific

The participants expressed the view that parental input to the review process should be optional and flexible; some parents did not wish to be involved initially but to have the option to contribute to the review or learn about its conclusions later. Some participants felt they may not have been able *“to handle it”* immediately due to the initial shock of the death. Others felt that it was important to carry out the review soon after the event so that it was *“fresh”* in the healthcare professionals’ minds. Participants felt that what they would have said straightway after the death would be different to how they felt about it now, months or over a year later.

*‘I wanted to know the medical stuff straight away … but emotionally I would have had different things to say about the care I had a year later.’*

Participants understood that there may be practical time limits to the feedback process however they recognised that there could be potential to revisit the case in the subsequent pregnancy. Continuity of care by the healthcare team was highlighted as an important factor to enable this to happen. However, one participant felt *“hesitant”* about revisiting the case during a subsequent pregnancy as it could exacerbate anxiety and have negative effects on the fetus.

During the focus group discussion participants were given a formatted letter that could initially be sent to parents following their pregnancy or neonatal loss. This letter has been developed by the Sands and DoH task group. They were asked about their views of the letter and its contents and about suggestions of ways to improve the letter. Participants thought that brief circumstance-specific information about the review should be included in discharge packs and discussions. Many participants felt that the letter should be personalized to their individual circumstance for example if the letter should be different depending on the circumstances of the loss, e.g. a letter regarding a stillbirth should differ from one that deals with a neonatal death.

*‘We have got neonatal deaths, stillbirths terminations, does this letter cover all those eventualities or do we need a letter that is slightly tailored to each circumstance?’*

Parents believed that some direction as to how parents might contribute to the process (e.g. example questions, a framework with subsections) would be useful, alongside an opportunity for free text input.

*‘You could frame that review process down into subsections, so it was not just about questions and answers about why your baby died, I think if you have got other questions and you’re talking about the care from the hospital and talking about communication.’*

Furthermore, several participants felt prior explanation of the proposed letter by a member of the healthcare team would be beneficial prior to discharge.

### Inclusive

Participants believed the review process should capture both the clinical and emotional aspects of each case. For individual cases, such as a termination for fetal congenital anomaly, treatment may not have been different but emotional care could have been improved.

*‘We understand why he is there, we understand what has gone wrong and understand why he has died… So it is more about the emotional side isn’t it?’*

The overall opinion of the focus group discussion was that the review should have a whole team care approach by giving rise to important lessons learned for not just obstetric and/or neonatal care but also community care.

*‘I did get a home visit from my GP but that was about it. So I would say the hospital did very well but then it stopped and that was where the care really needs to be looked at.’*

One parent wanted to have assurance that obstetricians, midwives, neonatologists and community staff discussed the case together.

*‘One of the key things for me, is also knowing that the different pathways … will get together and decide whether there was anything that we should’ve done differently… there are lots of people that are in your care.’*

Continuity of pathways of care emerged as a recurring sub-theme within the analysis. This need for continuity of care and communication was felt to be required not only locally within hospitals but regionally.

*‘Babies can go between lots of different hospitals … I have never got an answer to in years; is how much doctors actually talk to each other.’*

### Positive process

Participants commented that the review should include positive aspects of care and comments for individual or team excellence.

*‘It was just the consultant appointment and there wasn’t really an opportunity to say anything positive about the experience you know with regards to care or anything like that.’*

Parents felt that getting answers would help to alleviate self-blame and reassure them that there is accountability following the death of a baby.

*‘Being in a vacuum for nine weeks that was really hard because during that time you beat yourself up, blame yourself, make reason why it was your fault.’*

## Discussion

The PARENTS study investigated parental views about the current perinatal mortality review (PNMR) process and their thoughts on parental involvement in South West England. We found that parents felt they should be involved in PNMR, but both the timing and the method should be flexible and adaptable to their individual needs. Parents expressed the view that the process should be open and transparent, and emphasised the need for an inclusive and positive approach to both medical and emotional aspects of care.

### Strengths and limitations

This is the first-time parental opinion has been formally investigated in-depth on the PNMR process. We are not aware of any comparable studies within research area, but the key themes and sub-themes described reflect the wider literature on parents’ experience of stillbirth [3, 13, 14]. A systematic review published in 2016 found that parents would appreciate a healthcare system that provides support following discharge from hospital and also a follow up appointment that might resolve uncertainty [4].

For this study, we recruited a sample of women and their partners with a range of experience of pregnancy or neonatal loss. Although we have investigated the views of a diverse sample of parents in South West England, parents in other parts of the UK may express different views. Future research might be necessary, particularly with bereaved parents with diverse cultural, ethnic, and religious backgrounds.

Furthermore, we noted parents did not discuss the medico-legal implications of parental involvement in the PNMR process. This was perhaps because of this not being at the forefront of parents’ minds. Further research should explore healthcare professionals’ and stakeholders’ perceptions of parental involvement and what medico-legal implications this might entail. The medico-legal implications may also differ from country to country and therefore consideration needs to be made to this variation when interpreting any future findings.

We have been able to elucidate an aspect of perinatal death and the related hospital processes that had not been explored in depth before, yet was identified as critical for future health service improvement. Crucially, we explored this important issue together with a bereaved parent co-investigator who was integral to the study design, implementation and analysis of data.

### Interpretation and comparison to the literature

Mounting evidence shows that care at and around the time of perinatal death can positively influence outcomes for parents [2, 13]. In other areas of healthcare, an inclusive approach with patients as active participants in their care has been shown to improve patient satisfaction and health care quality [15–20]. Involving patients could play a role in improving future patient safety [21], because patients and families have a unique viewpoint and may be able to highlight errors unknown to the hospital and may be useful in improving future care [22]. Involving patients in understanding the sequence of traumatic and potentially life changing events they have experienced may help in the healing process [23]. The same principle might apply to maternity care for bereaved parents: their involvement might be similarly beneficial. However, it was important to understand the parents’ wishes first, before embarking of further research to explore the feasibility and acceptability of options for parental involvement.

## Conclusion

Evidence from a large focus group discussion undertaken with a diverse purposeful sample of bereaved parents showed that parents were largely unaware that a review of their child’s death took place, and found it distressing that they were not involved or kept informed. Parents were consistently in favour of an optional opportunity to contribute information, and would welcome a flexible system that could provide them with feedback, outcomes and lessons learned following the review. Further research is necessary to explore how to design and test a process that is standardised yet responsive, feasible and useful to parents and staff alike.

## Additional files

Additional file 1: PARENT focus group schedule. (DOCX 16 kb)

Additional file 2: Additional parental quotes. (DOCX 16 kb)

## Abbreviations

DoH: Department of health
MBRRACE-UK: Mothers and babies: reducing risk through adults and confidential enquiries across the UK
PARENTS: Parents’ active role and engagement in the review of their stillbirth/perinatal death study
PNMR: Perinatal mortality review
Sands: Stillbirth and neontal death charity

## References

[1] MBRACE. Perinatal mortality surveillance report 2013. https://www.npeu.ox.ac.uk/mbrrace-uk/reports. Accessed 02 July 2015. [Internet]. Available from: https://www.npeu.ox.ac.uk/mbrrace-uk/reports

[2] Heazell AEP, Siassakos D, Blencowe H, Burden C, Bhutta ZA, Cacciatore J (2017). Stillbirths: economic and psychosocial consequences. Lancet [internet]. Elsevier.

[3] Burden C, Bradley S, Storey C, Ellis A, Heazell AEP, Downe S (2016). From grief, guilt pain and stigma to hope and pride -- a systematic review and meta-analysis of mixed-method research of the psychosocial impact of stillbirth. BMC Pregnancy Childbirth [Internet].

[4] Ellis A, Chebsey C, Storey C, Bradley S, Jackson S, Flenady V (2016). Systematic review to understand and improve care after stillbirth: a review of parents’ and healthcare professionals’ experiences. BMC Pregnancy Childbirth [Internet].

[5] NHS England. NHS Outcomes Framework 2013/2014 [Internet]. Available from: https://www.gov.uk/government/uploads/system/uploads/attachment_data/file/213055/121109-NHS-Outcomes-Framework-2013-14.pdf

[6] Kirkup B. The Report of the Morecambe Bay Investigation [Internet].cited 2017 Feb 5. Available from: https://www.gov.uk/government/uploads/system/uploads/attachment_data/file/408480/47487_MBI_Accessible_v0.1.pdf

[7] Robert Francis. The The Mid Staffordshire NHS Foundation Trust Public Inquiry, 2013 [Internet]. [cited 2017 Feb 5] Available from: http://webarchive.nationalarchives.gov.uk/20140407084003/http://www.midstaffspublicinquiry.com/

[8] Manktelow BN, Smith LK, Prunet C, Smith PW, Boby T, Hyman-Taylor P, Kurinczuk JJ, Field DJ, Draper ES, on behalf of the MBRRACE-UK Collaboration. MBRRACE-UK Perinatal Mortality Surveillance Report, UK Perinatal Deaths for Births from January to December 2015. Leicester: The Infant Mortality and Morbidity Studies, Department of Health Sciences, University of Leicester. 2017.

[9] Draper ES, Kurinczuk JJ, Kenyon S. (Eds.) on behalf of MBRRACE-UK. MBRRACE-UK Perinatal Confidential Enquiry: Term, singleton, normally formed, antepartum stillbirth. Leicester: The Infant Mortality and Morbidity Studies, Department of Health Sciences, University of Leicester; 2015.

[10] Braun V, Clarke V (2006). Using thematic analysis in psychology. Qual Res Psychol Routledge.

[11] Tong A, Sainsbury P, Craig J (2007). Consolidated criteria for reporting qualitative research (COREQ): a 32-item checklist for interviews and focus groups. Int J Qual Heal Care [Internet].

[12] Siassakos D, Storey C, Davey L, Team the IS (2015). Stillbirth: public/patient involvement in sensitive research and research ethics. BJOG An Int J Obstet Gynaecol.

[13] Redshaw M, HJ RR. Listening to parents after stillbirth or the death of their baby after birth. NPEU, University of Oxford. 2014; Available from: https://www.npeu.ox.ac.uk/listeningtoparents

[14] Siassakos D, Jackson S, Gleeson K, Chebsey C, Ellis A, Storey C for the INSIGHT Study Group. All bereaved parents are entitled to good care after stillbirth: a mixed-methods multicentre study (INSIGHT). BJOG. 2017; https://doi.org/10.1111/1471-0528.14765. 10.1111/1471-0528.14765 PMC5763319 28758375

[15] Medicine I of (2001). Crossing the quality chasm; a new health systemf for the twenty-first century.

[16] G BP and R (2007). Bringing user exprience to healthcare improvement.

[17] C V (2010). Patient safety.

[18] Occlo JE, Fulop NJ. Developing a critical approach to patient and public involvement in patient safety in the NHS: learning lesson from other parts of the public sector? Health Expect. 2012;15:424–32. doi: 10.1111/j.1369-7625.2011.00695.x. 10.1111/j.1369-7625.2011.00695.x PMC5060634 21711471

[19] Langley GL, Nolan KM, Nolan TW, Norman CLPL (2009). The improvement guide: a practical approach to enhancing Oranizational performance 2nd edition.

[20] Etchegaray JM, Ottosen MJ, Burress L, Sage WM, Bell SK, Gallagher TH (2014). Structuring patient and family involvement in medical error event disclosure and analysis. Health Aff (Millwood) United States.

[21] Millman EA, Pronovost PJ, Makary MA, Wu AW (2011). Patient-assisted incident reporting: including the patient in patient safety. J Patient Saf United States.

[22] Iedema R, Allen S, Britton K, Gallagher TH (2012). What do patients and relatives know about problems and failures in care?. BMJ Qual &amp;amp; Saf.

[23] Practices. I for SM. Benefits and risk of including patients on RCA teams. 2008. Available from: https://www.ismp.org/newsletters/acutecare/articles/20080605_2.asp.

