# Supplementary material for: Learning from deaths: Parents’ Active Role and ENgagement in The review of their Stillbirth/perinatal death (the PARENTS 1 study)
Source: BMC Pregnancy Childbirth. 2017 Oct 2;17:333. doi: 10.1186/s12884-017-1509-z (PMC5625604; doi:10.1186/s12884-017-1509-z)
Supplement: Supplementary file 1 — PARENT focus group schedule. (DOCX 16 kb) [file 12884_2017_1509_MOESM1_ESM.docx]

PARents' ENgagement in Their Stillbirth review (PARENTS) Project

Interview schedule for Focus Group with parents who have experienced perinatal bereavement with the aim of exploring their perceptions & expectations of the perinatal mortality review process. How would they like to contribute & how would they like to receive feedback?

Focus group participants: There will be one focus group consisting of Bristol Sands & other bereaved parents who were (purposively) approached by CS. Each parent has had experience of perinatal bereavement at least 6 months or more ago. Approximately 12 participants (Mothers & Fathers) have been approached.

Facilitators: DS, CS, FY, FJ will conduct the focus groups in a private space away from any hospital site & agreed in advance with participants. CS is known to all participants & will be available to provide necessary support to those who experience emotional difficulties. The focus group will run for approximately 2 hours.

0 – 20 minutes: Welcome & introductions

Introductions from facilitators (including who we are & our roles)

Brief explanation of the aims of the focus group & rules for the discussion (e.g. confidentiality, respect for the circumstances of each participant & the choices they made about their baby/babies, ok to cry etc).

Ask participants to introduce themselves together with brief explanation of the circumstances surrounding their baby’s death.

Any questions?

20 – 40 minutes: Discussion 1 – Current process

Participants will be asked to consider what they believe currently happens when a baby has died & then to discuss the accuracy of their perceptions.

- Are they aware that there is a formal process to review each death?
- What do they think is the purpose of reviewing cases?
- Who do they think attends?
- How long do they think is spent discussing each case?
- How long do they think it takes before each case is reviewed?
- What do they think happens as a result of the process?

40 – 60 minutes: Discussion 2 – What could be done differently

Participants will be asked to consider the current processes, think about how it might be improved & discuss whether they think bereaved parents might have a valuable contribution to make

- Do they think it is appropriate for families to be informed of the process?
- How & when do they think this should happen, e.g. prior to discharge from hospital?
- How & when do they think it would be most appropriate to be contacted to see whether they would like to contribute?
- Should it be opt in or opt out?
- Do they think it preferable to have a face to face discussion with consultant (e.g. at the same time as their follow up appt) so that questions with simple answers (e.g. glass of wine at 8 weeks) can be put to rest & attention be spent on more complex questions?
- If not in person, would it be preferable to have a discussion over the phone, by email or by letter?
- Do they think there should be a standardised form to complete to ensure equity or is this too impersonal?

60 – 80 minutes: Discussion 3 – Receiving feedback

Participants will then be asked to consider how & when they might wish to receive feedback following the review process & how this might fit in with the current practice of follow up with a consultant

- At what stage do they think it would be appropriate to receive feedback after the review?
- How would they feel about an additional appointment, a phone call, email or letter?
- Would this opinion differ if the review had highlighted new & sensitive information that may require careful discussion?
- If by letter, how would they feel about a standard template or would they prefer it to be more personalised?

80 – 100 minutes: Discussion 4 – Sands/DoH letter templates & information

Participants will be provided with copies of the proposed letter templates & patient information & be asked to comment on them in light of previous discussions

- What do they think of the content & style of the letters?
- What is good?
- What could be improved?
- Can they envisage any problems doing this by letter rather than in person?
- What do they think about having a named contact?
- Who do you think the named contact should be (midwife, nurse, Consultant)?

100 – 120 minutes: Summary

Facilitators will summarise the main points of the discussions & ask whether there are any other issues to discuss before the focus group ends.

- Recap what has been discussed
- Re-iterate the aims of the focus group & what the facilitators hope to achieve as a result (next steps)
- Give participants an outline of what to expect next (e.g. we will write to them with results)
- Assure them of confidentiality
- Thank them for their time
