# Supplementary material for: Learning from deaths: Parents’ Active Role and ENgagement in The review of their Stillbirth/perinatal death (the PARENTS 1 study)
Source: BMC Pregnancy Childbirth. 2017 Oct 2;17:333. doi: 10.1186/s12884-017-1509-z (PMC5625604; doi:10.1186/s12884-017-1509-z)
Supplement: Supplementary file 2 — Additional parental quotes. (DOCX 16 kb) [file 12884_2017_1509_MOESM2_ESM.docx]

**Additional parental quotes from the PARENTS 1 Study**

| **Overarching Theme** | **Sub Theme** | **Quotes** |
| --- | --- | --- |
| **Transparency** | **Awareness of perinatal mortality review process** | ‘I honestly didn’t know there was one’  ‘Because she had been in hospital for so long I knew that there was some sort of process they went through…. so we knew that something had happened but we weren’t necessarily part of it’  ‘I did know that there was a review because X told me there was… but I don’t know what they discuss or what happens as a result of that’  ‘We had a relatively brief consultant appointment afterwards but no I wasn't aware of any review’ |
|  | **Parental reassurance** | ‘I don't want someone to lose their baby in Yorkshire and go through what I’ve been through if it could be stopped’ |
|  | **Lessons learned & accountability** | ‘Some of the people that come to the support groups and the stories they tell of the care they've received is pretty diabolical to be honest so you know anything that would stop those people having their experiences can only be a good thing’  ‘What you always hope is that babies that come after you will be better treated’  ‘We should know that we have lost our children and that other children aren't dying for those same reasons’  ‘You feel like why do you have to have death before something’s sorted’  ‘The processes and the grief you’re going through and its sort of at the time you’re trying to get sort of clues or an answer sometimes it just drags on too long and you just let go of those questions because it drags on too long’  ‘You question how much does THAT doctor get to know what THOSE doctors thought when they discussed what they wanted to do. And that is a big thing for me, and something I have never got an answer to in several years, is how much doctors actually talk to each other’ |
| **Flexible yet specific** | **Difference between medical & emotional care** | ‘I want to ask questions about my son’s care but I would like to feedback about the amazing experience we had with the hospital staff and care that we received, as a, as parents’  ‘If I were to get this through and it said ‘ohh let us know your questions’, I’d be like ‘well I haven’t got any questions’ but actually I could probably feedback a lot of other stuff, to do with our care and with our son’s care, and not questions about his medical, the circumstances’ |
|  | **Timing of the review process** | ‘If I think back and if I try to remember me at that moment, I wouldn't have been able to handle it or interact with it in a way that might be useful’  ‘What I would’ve said six weeks after it happened was, was very different to how I feel about it now’  ‘I think there needs to be that flexibility in the process for the parents who walk away, under their own choice, like ‘fine that’s closed, you do the review’ … but then six month later they change their minds, they need to be able to come back’ |
|  | **Open door policy** | ‘What we’ve actually touched on there, is actually very, very straightforward.. it’s an open policy, where everyone.. this review, or these reviews happen and nobody knows.. And all we are saying here is.. give people the choice…Make it more transparent’  ‘It needs to be an open process. This will happen, we’re sorry you’ve lost your son/daughter, this will happen. Do you feel that you want to be involved and want to know the outcome, want to speak to someone before, after, during’ |
| **Inclusive** | **Continuity of care** | ‘I have an outstanding memory… of being in the foyer waiting for my husband to bring the car around and feeling like, when I walk through that door something was going to be cut. You know the connection and that's where the care stopped’  ‘It wasn't clear, it what the process was and what the time process was and who you go to to chase if you needed to’  ‘The X had a bereavement midwife but I think she worked about 5 hours a week and most of that she was in sessions so it was really hard to get hold of her and she was one person in quite a big city’  ‘It really does get you down having to keep going back to the start and explaining why you’re there’  ‘For me I’d like to know who that person is. Because it’s all very well in having a name, but are they chaplain, the cleaner, you know, you kind of want to know who you’re talking to’ |
